# Enhancing NMDA Receptor Function: Recent Progress on Allosteric Modulators

**DOI:** 10.1155/2017/2875904

**Published:** 2017-01-09

**Authors:** Lulu Yao, Qiang Zhou

**Affiliations:** School of Chemical Biology and Biotechnology, Peking University Shenzhen Graduate School, Shenzhen 518055, China

## Abstract

The N-methyl-D-aspartate receptors (NMDARs) are subtype glutamate receptors that play important roles in excitatory neurotransmission and synaptic plasticity. Their hypo- or hyperactivation are proposed to contribute to the genesis or progression of various brain diseases, including stroke, schizophrenia, depression, and Alzheimer's disease. Past efforts in targeting NMDARs for therapeutic intervention have largely been on inhibitors of NMDARs. In light of the discovery of NMDAR hypofunction in psychiatric disorders and perhaps Alzheimer's disease, efforts in boosting NMDAR activity/functions have surged in recent years. In this review, we will focus on enhancing NMDAR functions, especially on the recent progress in the generation of subunit-selective, allosteric positive modulators (PAMs) of NMDARs. We shall also discuss the usefulness of these newly developed NMDAR-PAMs.

## 1. Introduction

NMDARs belong to the L-glutamate family, and they play important roles in synaptic transmission, synaptic plasticity, and experience-dependent refinement of synaptic connections during development [1, 2]. Their excessive activation or underactivation is proposed to contribute to the genesis or progression of various brain diseases, including stroke, schizophrenia, depression, and Alzheimer's disease [1, 3–5]. Past efforts in targeting NMDARs for therapeutic intervention had been focused on inhibiting these receptors with only limited success [6–8].

NMDARs are regarded as coincidence detectors because of their ligand-gated and voltage-gated properties that its activation requires both binding of glutamate and coagonist (glycine or D-serine) and postsynaptic depolarization. In addition, NMDARs contain several regulatory sites sensitive to polyamines, Zn^2+^, protons, and glutathione [1, 9]. The X-ray crystal structure of the NMDAR shows massive protein complexes, and each complex is composed of four subunits, which contains amino-terminal domain participating in assembling and modulation; a transmembrane domain forming an ion-channel pore; c-terminal domain involving in the trafficking of receptors and coupling to intracellular signaling molecules; and a ligand-binding domain binding agonists [10, 11]. NMDARs are composed of subunits from seven homologous genes, GluN1, GluN2A–GluN2D, and GluN3A-GluN3B. NMDARs are diverse in subunit composition, biophysical, and pharmacological properties, interacting partners and subcellular localization. Among these subunits, the four GluN2 (A–D) subunits are major determinants of the functional heterogeneity of NMDARs [12]. Different spatiotemporal expression profile is also a prominent feature of NMDARs. GluN2B is the dominant subunit at early age and reaches its peak expression in the first postnatal week, while GluN2A is most abundant in the adult brain in rodents. During postnatal brain development, an activity-dependent switch from GluN2B to GluN2A occurs. Synaptic NMDARs mainly contain diheteromeric GluN1/GluN2A and triheteromeric GluN1/GluN2A/GluN2B NMDARs at excitatory synapses on excitatory neurons. The percentage of triheteromeric NMDARs are estimated between one-third and two-thirds of total NMDARs [13–16]. Perisynaptic and extrasynaptic sites are enriched in GluN2B-containing receptors which are considered by some to trigger excitotoxicity and cell death when excessively activated [9]. Different types of neurons may express somewhat different combination of NMDAR subunits. While GluN2A and GluN2B subunits are highly expressed in the excitatory neurons, GluN2C and GluN2D subunits are more concentrated in the inhibitory GABAergic neurons [17, 18].

## 2. Enhancing NMDAR Functions

### 2.1. The Need to Enhance NMADR Functions

Proper development and refinement of neural circuit require the adequate function/activity of NMDARs. This can be understood as NMDARs are required to support synaptic plasticity mostly on the excitatory neurons [19]. On the other hand, it has been increasingly recognized that NMDARs on the GABAergic inhibitory neurons contribute to second-by-second synaptic transmission and hence excitation of these inhibitory neurons. As a result, reduced function of NMDARs on these inhibitory neurons may hinder their physiological functions and lead to the imbalance between excitation and inhibition [20–27].

### 2.2. NMDAR's Role in the Certain CNS Diseases

Most NMDAR-targeting pharmacological agents that have been tested in the clinical trials are nonselective in that they do not distinguish between NMDAR subunits. These broad spectrum NMDAR inhibitors, such as dizocilpine (MK-801), usually cause certain serious side effects including psychosis, memory impairment, and neuronal cell death. The majority of past efforts have been on generating inhibitors of NMDARs, for indications such as stroke, traumatic brain injury, and depression [29–31]. Ketamine has shown great promise in treating treatment-resistant depression with fast onset [32–34], although whether it is doing so via blocking NMDARs has been challenged recently [35]. Interestingly, rapastinel (also named GlYX-13) has shown antidepressant as an adjunctive therapy for treating depression [36]. GlYX-13 acts as a selective, weak partial agonist of the glycine site on the NMDARs. Unlike ketamine, GLYX-13 does not elicit psychotomimetic side effects. Recent evidence showed that via modulating NMDARs GLYX-13 leads to an increase in mature dendritic spines and a persistent reduction in the threshold for future induction of LTP [37–39]. In addition, another likely more potent drug NMDAR enhancer, sarcosine, a glycine transporter-1 (GlyT-1) inhibitor, was shown to improve the depression-like behaviors and symptoms [40, 41].

It is noteworthy that the development of NMDAR blockers for stroke has been met with failure in clinical trials, likely because the elevation in glutamate concentration during stroke is short-lasting (about half an hour after stroke onset) and hence NMDARs do not have time to act (most stroke patients do not get treated for at least a few hours after stroke onset). In addition, inhibiting NMDAR activation is likely to hinder the recovery process after stroke [42].

Certain evidence showed that both mRNA and protein levels of NMDARs are reduced in AD brain and AD model, suggesting hypofunction of NMDAR in the pathogenesis of Alzheimer's disease [43]. However, the level of NMDARs at a given synapse is reduced with AD progression which has not been demonstrated directly. Shankar et al. suggested that A*β* oligomers decreased the density of dendritic spines, NMDAR-mediated calcium influx, and internalization of synaptic NMDARs [44, 45]. Moreover, APP or ApoE4 mutant mice exhibit a decrease in NMDAR-mediated synaptic responses and impaired LTP; NMDAR hypofunction with advanced age is closely tied to redox state in the CNS [46]. However, in targeting NMDARs, only memantine has been approved by the FDA currently. Memantine is a NMDAR blocker and is thought to be neuroprotective by blocking excitotoxicity. It can be used for the treatment of moderate to severe AD, and recent studies demonstrated that memantine shows benefits in improving cognition, behavior, and daily living in AD patients [47, 48].

Substantial pharmacological, genetic, and biochemical evidence show that NMDAR hypofunction may have key contributions to the genesis of schizophrenia. For example, NMDAR antagonists, such as ketamine and phencyclidine (PCP), can induce schizophrenia-like phenotypes (including positive, negative symptoms, and cognitive deficits) in healthy individuals and exacerbate such symptoms in schizophrenic patients [49–52]. Large genome wide association study of schizophrenia implicated numerous genes involved in glutamatergic transmission, such as genes encoding the GluN2A subunits and serine racemase [53, 54]. Postmortem brain samples from schizophrenic patients also suggested reduced NMDAR function. These observations support the on-going efforts to enhance the function/activity of NMDARs. When enhancing NMDAR function, caution has been taken to avoid excessive activation which likely leads to excitotoxicity. In this regard, targeting coagonist at the glycine binding site or using NMDAR-PAMs may be a safer therapeutics option [55].

### 2.3. Glycine Binding Site Coagonists as NMDAR Enhancers

Glycine binding site has attracted attention of many scientists as a potential target for safely elevating the activity of NMDARs. Glycine binding site activation is obligatory for NMDAR opening [56, 57]. Glycine and D-serine are considered to be the two main endogenous coagonists. Functionally, glycine (or D-serine) binding increases the recovery rate for NMDARs and hence prolongs the duration of NMDAR EPSPs [58]. It can enhance the affinity and efficacy of glutamate binding on GluN2 subunit of NMDARs [59]. Electrophysiological results showed that both of the above actions can enhance NMDAR responses [60, 61]. Animal studies have shown that these coagonists and related modulators attenuated deficits in working memory and locomotor activity and reduced the deficits in prepulse inhibition [62, 63]. In recent years, a few clinical trials have shown efficacy of NMDAR enhancers in schizophrenia, such as DCS, sarcosine, and sodium benzoate. Participants receiving DCS showed enhanced potentiation of neural responses and cognitive performance [64, 65]. Sodium benzoate, as a d-amino acid oxidase inhibitor, significantly improved the PANSS total score and neurocognition in chronic schizophrenia patients [66] (ClinicalTrials.gov NCT00960219.)

Although some clinical trials show that daily administration of a large quantity of exogenous glycine or D-serine alone, as well as partial coagonists D-cycloserine or using these coagonists as an adjunct to atypical antipsychotic treatment, could result in an improvement of cognitive function and attenuate negative symptoms and positive symptoms in schizophrenia patients [3, 67, 68], other clinical trials had not produced positive outcomes; typically efficacy was seen in small trials while no efficacy was reported in larger trials [69, 70]. These observations suggest that NMDAR hypofunction may occur only in a subpopulation of schizophrenia patients, although how to identify this subpopulation is currently unknown. NMDAR modulators/enhancers are expected to be most effective in individuals exhibiting poor premorbid function, slow and incomplete response to antipsychotic agents, and relatively generalized nature of neurocognitive dysfunction [71]. The inconsistency between clinical trials using glycine may be explained by difference in the saturation of the glycine binding site, in that glycine will only be effective when the site is unsaturated. Whether the glycine binding site is saturated is still a matter of debate. Brain slice studies showed that the glycine site was saturated in the cerebellum but not in the PFC, hippocampus, and hypoglossal nucleus [72]. In vivo evidences also showed nonsaturation in the PFC [73, 74]. How to resolve the inconsistent clinical trials and identify effective future therapy is a major challenge. Identifying biomarkers that are directly linked to or associated with the underlying pathology of schizophrenia will be a major step forward in the individualized diagnosis and therapy.

Among the reagents targeting the glycine binding site, glycine transporters (GlyTs) have attracted efforts from many researchers for their key roles in regulating glycine concentration in the vicinity of NMDARs [75, 76]. Both GlyT-1 and GlyT-2 belong to the sodium-dependent solute carrier family 6. GlyT-1 and GlyT-2 show different regional and subcellular expression patterns. while GlyT-1 is expressed in most regions of the brain, mainly in glial cells and in presynaptic neurons, the expression of GlyT-2 is located on glycinergic neurons in brain stem and spinal cord [77–80]. Two GlyT-1 inhibitors have attracted a lot of attention. One is sarcosine, as mentioned above, an intermediate metabolite of glycine metabolism. It was shown to be beneficial for short-term treatment in acutely ill and chronically stable schizophrenia [81, 82], and in major depression [40]. The other GlyT-1 inhibitor, Bitopertin developed by Hoffmann-La Roche, has been tested in clinical trials. Bitopertin reached phase III clinical testing for treating negative symptoms or positive symptoms in schizophrenia. These trials were halted due to lack of efficacy in improving negative symptoms, which is the primary endpoint for these trials [53]. However, in this year, a clinical trial in Japan population showed that Bitopertin can improve the “negative” and “suboptimally controlled” symptoms, and Bitopertin showed good safety and tolerated [83]. Bitopertin remains hopeful for treating schizophrenia.

Some studies reported that glycine primarily targeted the extrasynaptic NMDARs while D-serine regulates the activity of synaptic NMDARs [84]. If so, it suggests that elevating glycine may preferentially enhance the activation of extrasynaptic NMDARs which might not be sufficient or appropriate to modulate functions in schizophrenia patients. Furthermore, Matsui et al. suggested that D-serine was up to three times more potent than glycine on NMDA receptors [85]. Following this logic, augmenting synaptic NMDAR function by elevating D-serine level may be a more effective therapeutic option [66, 84].

Overall, targeting the glycine binding site enhances NMDAR-mediated response which may avoid excitoxicity and neuronal degeneration compared to targeting the glutamate binding site. In addition, reagents targeting the glycine binding site can readily cross the blood brain barrier which is convenient for clinical use. Many preclinical and clinical studies have shown that targeting the glycine binding site can be readily achieved, but certain issues need to be taken into consideration. First, long-term treatment with glycine binding site enhancer is required. Although acute efficacy of targeting glycine site has been reported in some clinical trials, there is no clear evidence for benefits after long-term treatment. On the other hand, there have been reports that chronic D-serine treatment promoted NMDAR internalization, chronic D-cycloserine treatment resulted in NMDAR desensitization [86], and chronic glycine treatment may preferentially act on extrasynaptic NMDARs [53]. Second, some studies suggested that excessive activation of glycine binding site may contribute to the excitotoxicity in neurodegenerative diseases, such as amyotrophic lateral sclerosis (ALS) [87]. If this is the case, this suggests a potential on-target toxicity which may prevent long-term dosing of such compounds. Third, some patients treated with glycine showed recurrent gastrointestinal upset [88]. Large doses of D-serine treatment may be associated with renal toxicity [89, 90], although clinical trials have not shown kidney dysfunction [91]. These observations suggest potential side effects associated with targeting the glycine binding site. Taken all together, although the rationale for targeting glycine binding site is clear and suitable drug-like candidates are available, this target may not be ideal for clinical applications, especially long-term.

### 2.4. NMDAR-PAMs

In addition to altering the level of coagonists (such as glycine) to enhance NMDAR activation, allosteric modulation is another option for enhancing NMDAR functions. Allosteric modulators have been regarded as the next generation CNS therapeutics based on the discovery and clinical success of benzodiazepines which enhance the GABA_A_ receptors in an allosteric manner [92]. By definition, allosteric modulators have little or no effect in the absence of agonists (such as glutamate or GABA) but enhance (positive, PAMs) or reduce (negative, NAMs) the responses in the presence of agonists. This nature ensures that PAMs or NAMs only exert their actions physiologically at the right location (the active synapses) and at the right time (during receptor activation). Hence, PAMs/NAMs should have much reduced undesirable effects caused by excessive activation or inhibition. Typically, PAMs/NAMs do not bind at the agonist-binding sites of the targeted receptors. In addition, the saturability of allosteric modulators can decrease the risk of overdose, and PAMs/NAMs have high receptor selectivity since the binding sites of allosteric modulators are less conserved [6, 93].

#### 2.4.1. Early NMDAR-PAMs

Besides endogenous NMDAR-PAMs, such as histamine, ATP, spermine, Mg^++^, and neurosteroids, a few series of NMDAR-PAMs have been reported in the past years, including phenanthrene derivatives, naphthalene derivatives, and coumarin derivatives [94]. UBP compound is a family of agents targeting GluN1/GluN2 agonist-binding site of NMDARs. The subunit selectivity varies between compounds and either potentiation or inhibition has been observed. UBP512, a phenanthrene derivative, potentiated GluN2A-NMDARs with little or no effect on GluN2B-NMDARs and inhibited GluN1/GluN2C and GluN1/GluN2D NMDARs selectively. UBP551, a naphthalene derivative, potentiated GluN2D-NMDARs while inhibiting Glu2A-, Glu2B-, and Glu2C-NMADRs. The mechanism underlying the potentiation and inhibition is unknown [95, 96]. UBP714, a coumarin derivative, potentiated GluN2A-, GluN2B-, and GluN2D-NMDARs slightly [97]. CIQ, acting at the transmembrane domain, selectively potentiated GluN2C- or GluN2D-NMDARs [98, 99]. Chimeric receptor and point mutation studies demonstrated that the N-terminal domain, ligand-binding domain, and T592 of GluN2D played a key role in mediating the effect of CIQ [98]. However, whether these compounds are active or effective in a biological system has not been much tested.

#### 2.4.2. The GNE Series of NMDAR-PAMs

Recently, a Genentech group has reported a few series of GluN2A-selective PAMs and has done fairly substantial characterizations on these PAMs, including structural analysis of binding sites and functional activity [28]. The rationale for generating subunit-selective, direct enhancer of NMDARs is to optimally enhance the physic activation of NMDARs while minimizing pathological activation. Some studies indicate that GluN2A-containing NMDARs are important for synaptic plasticity [100, 101], and their functions are reduced in certain brain diseases such as schizophrenia, while excessive activation of GluN2B-NMDARs may trigger excitotoxicity in such conditions as stroke/ischemia and Alzheimer's diseases [102–105]. Thus it might be desirable to generate GluN2A-selective NMDAR-PAMs which do not activate GluN2B-NMDARs. These NMDAR-PAMs are generated first via a massive screening over 1.4 million compounds on GluN1-GluN2A NMDAR expressing HEK293 cells, followed by medicinal chemistry effort to improve their selectivity and potency. The reported PAMs are in the uM potency range (EC50), with varying degree of selectivity over AMPARs and subunits of NMDARs.

The reported GNE NMDAR-PAMs are very selective against GluN2A, with little or no potentiation of GluN2B, and with about 10-fold lower potency towards GluN2D. Since, in the adult, GluN2D is mainly expressed on the inhibitory neurons in the midbrain [106], the nonselective effects on these neurons have been limited on the general brain function. Most of the reported PAMs are also highly selective against the AMPARs. There is certain evidence that, in addition to diheteromeric form, triheteromeric form represents a significant fraction of total NMDARs and contributes to synaptic function and plasticity [14, 107]. These triheteromeric NMDARs are also potentiated by PAMs, although not as effective as the diheteromeric ones. The selectivity of PAMs against GluN2A-NMDARs was confirmed by a lack of effect in the GluN2A-KO mice.

How is this subunit selectivity achieved? Analysis of crystal structure of the dimer of GluN1-GluN2A ligand-binding domain in complex with GNE-6901 revealed that it binds to this region of the NMDARs. Further analysis identified V783 in GluN2A as a critical residue to endow the subunit selectivity of GNE NMDAR-PAMs. While V783 engages GNE-6901 via direct van der Waals contact, the corresponding residue F784 in GluN2B is much larger, protrudes to the binding sites, and may prevent GNE-6901 from binding to GluN2B-NMDARs. For the AMPARs, it appears that GNE-6901 acts as a silent allosteric modulator in that it can bind to the analogous site in the AMPARs but does not have any efficacy at this site. Thus, the selectivity against AMPARs is achieved via absence of efficacy rather than potency.

There are a few mechanisms that NMDAR-PAMs can potentiate the agonist-induced responses. One mechanism is by enhancing the potency of agonists which is exhibited as a leftward shift on the agonist concentration-response curve; that is, the same magnitude of responses can be obtained at lower agonist concentrations in the presence of PAMs. This situation may be especially useful or effective when the agonist is not saturated. Since NMDARs have two coagonists, glutamate and glycine, this increase in potency could be mediated by enhanced potency of glutamate, glycine, or both. GNE-8324 caused a significant increase in glutamate potency while GNE-6901 had much smaller effect; neither affected glycine potency. Another mechanism of PAMs is to prolong the activation of receptors, such as by reducing deactivation. Both GNE-6901 and GNE-8324 showed slowing of kinetics at saturating concentration of glutamate which is due to reduced deactivation, with GNE-8324 more pronounced than GNE-6901. Specifically, the potentiation by GNE-8324's effect had a much bigger dependence on the glutamate concentration than GNE-6901.

**Figure 1:**
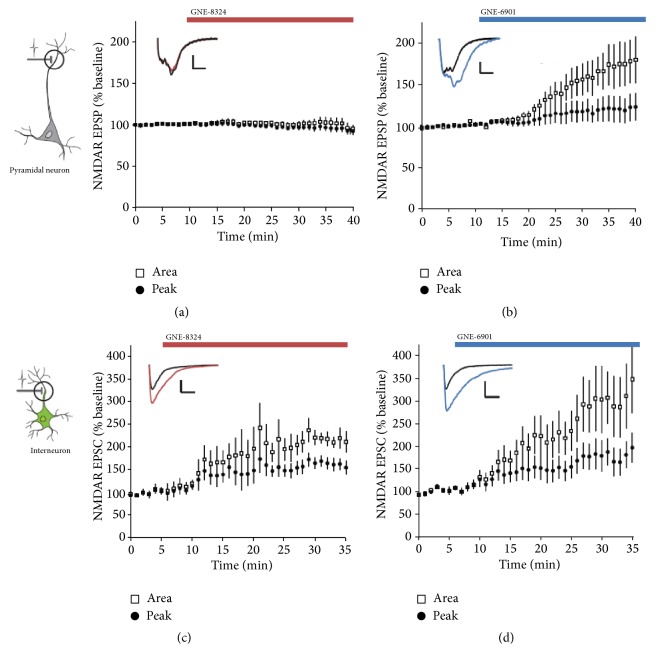
Cell type-specific effects of NMDAR-PAMs. (a) GNE-8324 has no effect on NMDAR EPSPs recorded from CA1 pyramidal neurons in acute brain slices. (b) In contrast, GNE-6901 showed robust potentiation on NMDAR EPSPs under the same condition. (c, d) Robust potentiation of both GNE-8324 and GNE-6901 on NMDAR EPSCs recorded in the inhibitory neurons in the hippocampus. Modified from [28].

A very interesting and important result of this study is that Hackos et al. had identified two types of NMDAR-PAMs [28], GNE-6901 that potentiated NMDARs on both excitatory and inhibitory neurons and GNE-8324 that only potentiated NMDARs on the inhibitory neurons (Figure 1). The mechanism underlying this differential potentiation is still unclear at this moment. One possibility is that the synaptic glutamate concentrations at the glutamatergic synapses on inhibitory neurons are much larger than that on the excitatory neurons. This interesting feature of GNE-8324 allows it to be used as a great tool to examine the effects of selectively enhancing inhibition (see below).

**Figure 2:**
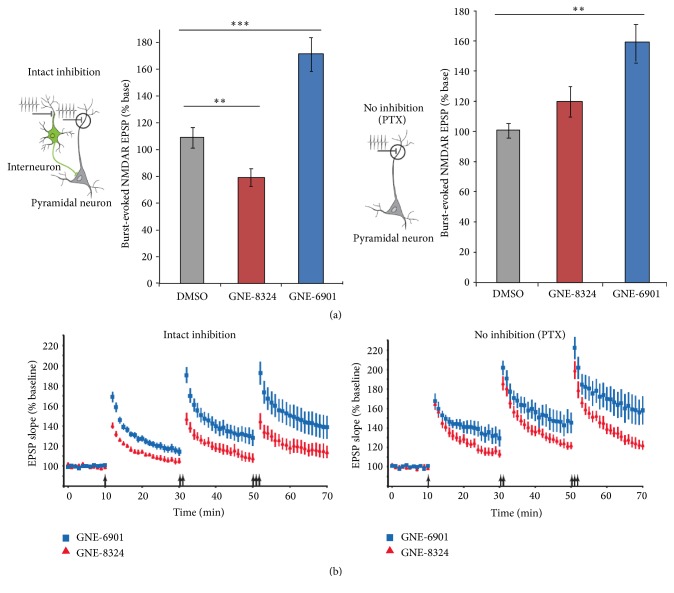
Differential effects on NMDARs underlie different modification of synaptic plasticity by GNE-PAMs. (a) NMDAR-mediated responses during TBS were calculated and showed differences between GNE-8324 and GNE-6901 which further depends on whether inhibition is present. With intact inhibition, GNE-6901 enhanced while GNE-8324 reduced NMDAR responses, compared to controls. When inhibition was absent, GNE-8324 showed a small, nonsignificant enhancement. (b) Consistent with the above effects on NMDAR responses, GNE-6901 enhanced LTP regardless of whether inhibition was present, while GNE-8324 enhanced LTP in the absence of inhibition but reduced LTP when inhibition was intact. Modified from [28].

NMDARs are required for the induction of long-term potentiation (LTP) of glutamatergic synapses on the excitatory neurons, and hence the PAMs of NMDAR are supposed to enhance LTP. This is certainly the case for GNE-6901, but not for GNE-8324 (Figure 2). To further understand how much effect the PAMs have on the activation of NMDARs during LTP induction (with theta burst stimulation, TBS), NMDAR responses from the pyramidal neurons during TBS were calculated from recordings. Interestingly, the magnitude of enhancement during TBS depended on whether inhibition was intact. When inhibition was intact (as seen under physiological conditions), a significant enhancement of NMDAR responses by GNE-6901 was seen; but for GNE-8324 there was a small but significant reduction. These results can certainly explain the differential effects of PAMs on LTP. On the other hand, when inhibition was blocked, the reduction in NMDAR responses by GNE-8324 was switched to a small and nonsignificant enhancement, indicating that activation of NMDARs on the inhibitory neurons is responsible for the reduced NMDAR responses on excitatory neurons during TBS. Smaller LTP may be beneficial in certain diseases, such as schizophrenia. An enhanced LTP has been reported in a mouse model of schizophrenia, which is likely caused by reduced inhibition [108]. Recently, Volgraf et al. have reported the synthesis of potent GluN2A-selective NMDARs PAMs, such as GNE-0723, which is highly brain penetrant. In addition to extensively testing in brain slices and on oocytes, GNE-0723 has been shown to be suitable for in vivo testing of GluN2A-PAMs [109]. The future in vivo studies on NMDAR-PAMs will provide more definitive testing for their potential in drug development.

#### 2.4.3. Important Unresolved Issues

Although the GNE series of NMDAR-PAMs have provided many new insights into the functioning of NMDARs and their contributions to brain under both physiological and pathological conditions, we have only seen the tip of the iceberg. Here, we have listed three important issues that can be resolved by using these PAMs as tool compounds.

*(1) Excitoxicity: Is It Subunit-Dependent?* It is still debated whether excitotoxicity is directly related to or caused by the subunit composition or the subcellular locations of the NMDARs that are excessively activated during pathological conditions such as stroke. In such context, it has been proposed that either GluN2B-NMDARs or extrasynaptic NMDARs are responsible for inducing excitotoxicity [102, 103, 110, 111]. If GluN2A-selective PAMs can enhance NMDARs that do not contain GluN2B subunits in the absence of causing much excitotoxicity, then it can be argued that the subunit model is likely correct. However, it is not understood whether enhancing activation of physiological activity itself is sufficient to trigger excitotoxicity. In this regard, a control experiment using NMDAR-PAMs that can selectively enhance GluN2B-NMDARs will be critical. Thus, NMDAR-PAMs can be used as a tool compound to test the contribution of subunit composition in excitotoxicity.

*(2) Kinetics: How Much Does It Matter?* Although the ideal situation is that enhanced activation preserves the kinetics of the receptor response, the nature/mechanism of the current NMDAR-PAMs dictates that this wish may not easily be granted. When enhancement involves reduced deactivation and hence slowing of kinetics, the responses mediated by activation of NMDARs may affect the encoding and transmission of information. For example, if potentiation of NMDAR response is sufficient to trigger more spiking activity in neurons due to prolonged depolarization, the increased numbers of spikes per given input may alter the encoding of sensory information or degrade their temporal patterns. This scenario may not be a major issue for NMDARs on the excitatory neurons since their major role is enabling the induction of synaptic plasticity (which is mostly a threshold-triggering event) compared to the second-by-second transmission of information. However, for inhibitory neurons, things might be different since there is ample evidence that NMDARs on these neurons are involved in the integration of inputs and generation of spiking activity [112], although there is no direct test of whether the dynamics of NMDARs affect processing or synaptic transmission in these inhibitory neurons in vivo. Some researchers looked at the contribution of NMADRs to the functioning of fast-spiking interneurons in the above context and they concluded that there is very limited contribution since the density of NMDARs on these neurons is very low [112–116]. Again, these new GNE NMDAR-PAMs may provide a great opportunity to understand this issue and the best test is to compare information encoding and synaptic transmission in vivo in the absence and presence of NMDAR-PAMs.

*(3) Enhancing Inhibition: Is It Sufficient?* Quite a bit emphasis has been put on the contribution of inhibitory neurons to the genesis of various CNS diseases, such as schizophrenia and Alzheimer's disease [1]. What these studies appear to suggest is that inhibitory neurons are directly affected (such as having reduced synapse density or spiking) rather than the postsynaptic GABA receptors, at least at the early stage of the diseases. Thus, a direct test to this hypothesis, as well as a potential new way to treat such diseases, is to enhance inhibitory functions by enhancing the activity of the affected inhibitory neurons. So far this idea has not been tested due to the lack of reagents to directly and selectively enhancing the activity in the inhibitory neurons. Since GNE-8324 can selectively enhance the NMADRs on the inhibitory neurons, it can be used to test the above hypothesis and may thus provide some new insights into the contribution of inhibition to the genesis of certain brain diseases and the potential of enhancing inhibition as therapeutics.

## 3. Prospective and Future Research

From selectively inhibiting NMDARs to enhancing their functions, we have undergone a drastic change in our thinking about the nature and contribution of these important receptors to physiological functions and pathological processes. Although there have been a lot of excitements with the recent rapid progress in the generation of various series of distinct NMDAR-PAMs, we are only at the very beginning of a new era to use these compounds as tools to further understand the nature of NMADRs, brain functions, and whether enhancing NMDARs can be a viable option in treating brain diseases.
